# GDNF-independent ureteric budding: role of PI3K-independent activation of AKT and FOSB/JUN/AP-1 signaling

**DOI:** 10.1242/bio.20135595

**Published:** 2013-07-30

**Authors:** James B. Tee, Yohan Choi, Ankur Dnyanmote, Marvalyn Decambre, Chiharu Ito, Kevin T. Bush, Sanjay K. Nigam

**Affiliations:** 1 Department of Medicine, University of California, San Diego, La Jolla, CA 92093-0693, USA; 2 Department of Pediatrics, University of California, San Diego, La Jolla, CA 92093-0693, USA; 3 Department of Cellular and Molecular Medicine, University of California, San Diego, La Jolla, CA 92093-0693, USA; ‡ Present address: Department of Pediatrics, University of Calgary and Alberta Children's Hospital, Calgary, AB T3B 6A8, Canada; § Present address: Department of Integrative Biology and Pharmacology, University of Texas Health Science Center, Houston, TX 77225, USA; ¶ Present address: Departments of Pediatrics and Surgery, University of California, San Diego, La Jolla, CA 92103, USA; ** Present address: Department of Medicine and Clinical Science, Okayama University Graduate School of Medicine, Okayama 700-8558, Japan

**Keywords:** Ureteric bud, Kidney development, Wolffian duct, AKT, Jun-Fos, JNK signaling, ret, Fibroblast growth factor

## Abstract

A significant fraction of mice deficient in either glial cell-derived neurotrophic factor (GDNF) or its co-receptors (Gfrα1, Ret), undergoes ureteric bud (UB) outgrowth leading to the formation of a rudimentary kidney. Previous studies using the isolated Wolffian duct (WD) culture indicate that activation of fibroblast growth factor (FGF) receptor signaling, together with suppression of BMP/Activin signaling, is critical for GDNF-independent WD budding (Maeshima et al., 2007). By expression analysis of embryonic kidney from Ret^(−/−)^ mice, we found the upregulation of several FGFs, including FGF7. To examine the intracellular pathways, we then analyzed GDNF-dependent and GDNF-independent budding in the isolated WD culture. In both conditions, Akt activation was found to be important; however, whereas this occurred through PI3-kinase in GDNF-dependent budding, in the case of GDNF-independent budding, Akt activation was apparently via a PI3-kinase independent mechanism. Jnk signaling and the AP-1 transcription factor complex were also implicated in GDNF-independent budding. FosB, a binding partner of c-Jun in the formation of AP-1, was the most highly upregulated gene in the ret knockout kidney (in which budding had still occurred), and we found that its siRNA-mediated knockdown in isolated WDs also blocked GDNF-independent budding. Taken together with the finding that inhibition of Jnk signaling does not block Akt activation/phosphorylation in GDNF-independent budding, the data support necessary roles for both FosB/Jun/AP-1 signaling and PI3-kinase-independent activation of Akt in GDNF-independent budding. A model is proposed for signaling events that involve Akt and JNK working to regulate GDNF-independent WD budding.

## Introduction

Development of the mammalian (metanephric) kidney begins when the Wolffian duct (WD), a paired mesonephric organ in mammalian embryos, is induced by signals arising from adjacent metanephric mesenchyme (MM) cells to form a localized epithelial outgrowth known as a ureteric bud (UB). Growth and branching of the UB will ultimately give rise to the tree-like collecting system of the kidney from the connecting segment to its insertion into the bladder. Timely induction and proper growth of the UB is critical for the appropriate formation of the kidney as subsequent elongation and branching of this epithelial bud dictates renal architecture (i.e. spatial arrangement of nephrons via its induction of mesenchymal-to-epithelial transformation of the MM), a fundamental determinant of kidney function (Shah et al., 2004; Costantini and Shakya, 2006; Shah et al., 2009).

Glial cell-derived neurotrophic factor (GDNF), a member of the transforming growth factor beta (TGF-β) superfamily of growth factors, is the main soluble factor that induces formation of the UB from the WD by way of signaling through the Ret receptor tyrosine kinase and its co-receptor GFRα1 (Sariola and Saarma, 2003). GDNF-null mice are characterized by renal agenesis, dysgenesis, or hypogenesis (Moore et al., 1996; Pichel et al., 1996; Sánchez et al., 1996), while mice lacking either GFRα1 (Cacalano et al., 1998) or Ret (Schuchardt et al., 1994; Schuchardt et al., 1996) display similar phenotypes. Deletion of upstream mediators of GDNF expression, such as *Eya1*, *Pax2*, and *Gdf11*, also results in renal agenesis (Xu et al., 1999; Bouchard et al., 2002; Esquela and Lee, 2003; Li et al., 2003; Brodbeck and Englert, 2004; Shah et al., 2004). Nevertheless, up to one-half of GDNF, Ret, and GFRα1 knockout mice continue to form a UB for reasons that remain unclear (Schuchardt et al., 1994; Moore et al., 1996). In addition, ex vivo data support the notion that budding of the UB can occur in the absence of GDNF-Ret mediated signaling (Maeshima et al., 2007). FGF signaling and suppression of BMP/Activin appear to play a key role in GDNF-independent budding; a notion supported by both in vitro and in vivo data (Maeshima et al., 2006; Maeshima et al., 2007; Michos et al., 2007; Michos et al., 2010). Thus, while it is clear that GDNF signaling is an important promoter of UB outgrowth, it is also clear that this is not the only growth factor signaling cascade capable of regulating this process.

Here we have utilized a combination of ex vivo/in vitro wet lab perturbation and transcriptomic analyses of Ret^(−/−)^ early embryonic kidneys in an attempt to identify growth factor signaling cascades potentially important in GDNF-Ret independent WD budding. The data support a central role for both FosB/Jun/AP-1 signaling and PI3-kinase-independent activation of Akt in GDNF-independent budding. A model is proposed for growth factors and downstream signaling events regulating GDNF-independent WD budding.

## Materials and Methods

### Reagents

JNK inhibitor II, LY294002 (PI3K inhibitor), Akt inhibitor IV and recombinant rat FGF1 were from CalBiochem (EMD, San Diego, CA). Recombinant rat GDNF, FGF7, follistatin, and goat anti-GFRα1 were from R&D Systems (Minneapolis, MN). Fetal bovine serum (FBS) was from Biowhittaker (Walkersville, MD). DMEM/F12 was from Gibco (Invitrogen, Carlsbad, CA). Mouse anti-ZO-1 and mouse anti-E-Cadherin were from Zymed (Invitrogen). Alexa Fluor 488 or 594 secondary antibodies were from Molecular Probes (Invitrogen). All other reagents were from Sigma (St. Louis, MO).

### Isolation and culture of Wolffian ducts

Wolffian ducts (WDs) isolated from E13.5 Sprague-Dawley rat embryos (Harlan, Indianapolis, IN) were dissected free from surrounding mesonephric tissues such that a thin layer of intermediate mesoderm remained associated with the epithelial tube (Zhang et al., 2012). These so called “semi-clean” WDs were cultured on top of Transwell filters (0.4 µm pore size; Costar, Cambridge, MA) for up to 7 days in DMEM/F12 supplemented with 10% FBS in the absence or presence of various growth factors and/or inhibitors as indicated (Maeshima et al., 2007; Rosines et al., 2007; Choi et al., 2009; Tee et al., 2010).

### Microarray

Mice heterozygous for Ret in the 129/Sv background were mated to generate Ret knockout animals and wild-type controls. Embryos were genotyped (Schuchardt et al., 1994) and kidneys were visually inspected for the presence of a ureteric bud before processing for microarray analysis. Wild-type and mutant kidneys were lysed, total RNA was extracted (RNEasy Micro kit; Qiagen, Germantown, MD), processed and hybridized to the GeneChip Mouse Genome 430 2.0 microarray (Affymetrix) by the UCSD genechip core by as previously described (Choi et al., 2009; Tee et al., 2010). GeneSpring GX 11.5 (Agilent, Santa Clara, CA) was used to analyze fold-change data. Data was preprocessed by converting any value less than 0.01 to 0.01. Data was normalized per chip to the 50^th^ percentile. Data was normalized per gene to the median. Network/pathway analysis was performed using the Ingenuity Pathway Analysis (IPA, Ingenuity Systems, Redwood City, CA) plugin for GeneSpring (Choi et al., 2009; Tee et al., 2010).

### Immunohistochemistry

Isolated WD cultures fixed in 4% PFA for 1 hour at room temperature were processed for immunohistochemical fluorescent staining as previously described (Choi et al., 2009; Tee et al., 2010). Samples were visualized with a Nikon D-Eclipse 80i confocal microscope.

### Real-time quantitative PCR

Total RNA was extracted from WDs (RNAqueous-Micro RNA Purification kit; Ambion, Foster City, CA) and amplified into cDNA with the SuperScript III system (Invitrogen, Carlsbad, CA) with ∼100 ng of RNA per reaction. Primers for genes were generated using Primer Express 3.0 software (Applied Biosystems, Foster City, CA). Quantitative PCR was performed using Syber Green/Rox (Invitrogen) and Fast Real-Time PCR 7500 (Applied Biosystems). Cycle thresholds (Ct) values were normalized to GAPDH using the formula 2^(GAPDH−sample)^. Triplicate samples were analyzed and significant fold changes were determined using Student's T-Test.

### Small interfering RNA (siRNA)

On-TargetPlus Rat FosB siRNA was purchased from Dharmacon (Chicago, IL) with a target sequence of: CAUCAAGCCCAUUAGCAUU. On-TargetPlus non-targeting siRNA #1 (D-001810-01-05, Dharmacon) was utilized as a non-targeting mismatch control oligo. Isolated WDs were cultured on top of Transwells in the presence of DME/F12 supplemented with 10% FBS for four to six hours before transfection to allow for adhesion of the WDs to the membrane. DharmaFECT I (Dharmacon) was diluted to 3% in Opti-MEM (Gibco) and siRNA was diluted to 1 µM in Opti-MEM. Following separate 5 minute incubations at room temperature, the siRNA mixture was combined with the DharmaFECT I mixture to generate a final siRNA oligomer concentration of 500 nM. The mixture was gently mixed together at room temperature for 20 minutes and then then applied on top of the Transwell filter, directly in contact with the isolated WDs. 125 ng/ml GDNF and FGF1 were added to the media in the well below the Transwells and the culture was allowed to proceed for 48 hours.

## Results

The GDNF-ret signaling pathway, which induces the outgrowth of the UB from the WD, is perhaps the best studied pathway for kidney development and is sometimes considered essential for the first step in nephrogenesis. Nevertheless, a significant number (i.e. 20–50%) of knockouts of either GDNF or one its co-receptors (ret and GFRα1) undergo budding and form rudimentary kidneys (Schuchardt et al., 1994; Moore et al., 1996; Pichel et al., 1996; Sánchez et al., 1996; Schuchardt et al., 1996). Despite the fact that these kidneys are generally hypoplastic with a reduced capacity to undergo branching morphogenesis, the presence of even a rudimentary kidney indicates that UB outgrowth (the initiating event in metanephric kidney development) must have occurred even in the absence of canonical GDNF-ret-mediated signaling demonstrating the existence of an in vivo “bypass” pathway.

### Evaluation of gene expression reveals increases in the expression of a number of FGFs in the Ret^(−/−)^ tissue that undergoes budding

**Fig. 1.**
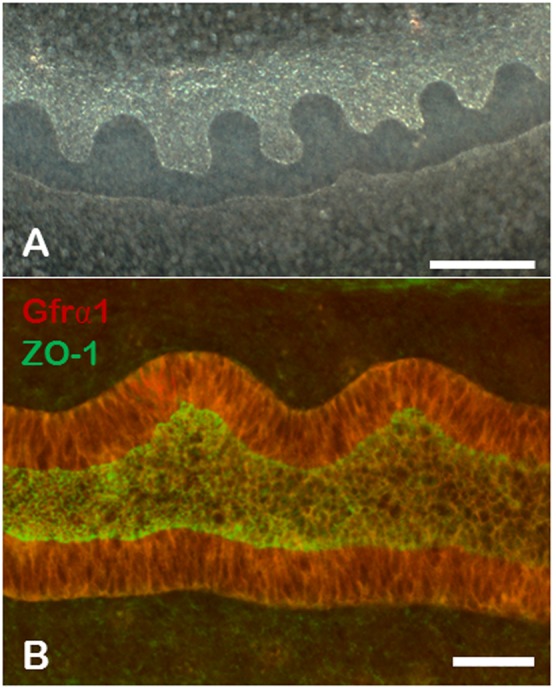
Wolffian duct budding could be induced ex vivo in the absence of GDNF. (**A**) Darkfield photomicrograph of isolated Wolffian ducts cultured for three days in the presence of 125 ng/ml FGF7 and 500 ng/ml of follistatin. Multiple buds can be seen. Scale bar: 200 µm. (**B**) Confocal photomicrograph of isolated WD cultured under GDNF-independent conditions and stained for GFRα1 (red) and ZO-1 (green). Scale bar: 25 µm.

This “bypass” pathway has been reconstituted in an in vitro isolated WD culture system and reliable GDNF/Ret-independent budding has been achieved with the exogenous addition of an FGF (i.e. FGF1 or FGF7) together with simultaneous inhibition of activin signaling with follistatin (Fig. 1) (Maeshima et al., 2007; Rosines et al., 2007; Choi et al., 2009; Tee et al., 2010). Although GDNF-independent budding will occur in cultures of the whole mesonephros, in order to limit potential extraneous signaling events, the epithelial WD is mechanically microdissected away from the majority of the surrounding mesonephric mesenchyme leaving all but a thin layer of mesodermal cells associated with the WD epithelial tissue (Maeshima et al., 2007; Rosines et al., 2007; Choi et al., 2009; Tee et al., 2010). Although the exact FGF remains unknown, a roughly analogous condition has been used to demonstrate GDNF-independent budding in vivo, where FGFs 7, 10 or a combination have been suggested as possible mediators of an in vivo GDNF-independent budding “bypass” pathway (Chi et al., 2004; Maeshima et al., 2007; Choi et al., 2009; Michos et al., 2010; Pitera et al., 2012).

**Fig. 2.**
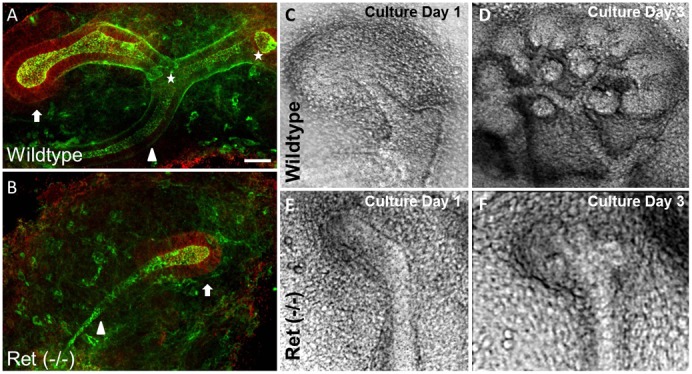
Comparison of Ret^(−/−)^ and wildtype kidney. (**A**,**B**) Confocal photomicrographs of embryonic mouse kidneys isolated from a wildtype (A) and Ret knockout (B) mouse. Stars indicate the points of bifurcation, arrows indicate localization of GFRα1 (red) and arrowheads indicate that portion of the ureteric bud external to the metanephric mesenchyme. (**C–F**) Phase contrast photomicrographs of E11.5 mouse kidneys cultured on top of Transwell filters with 10% FBS in DMEM/F12 for three days. (C,D) Ret^(+/−)^ kidneys underwent iterative branching morphogenesis and the formation of nephrons. (E,F) In contract, Ret knockout kidneys did not undergo iterative branching or mesenchymal-to-epithelial transformation. (A,B) Red  =  GFRα1; green  =  E-cadherin and ZO-1. Scale bar: 50 µm.

As described above, the appearance of a rudimentary kidney (albeit hypoplastic) in some Ret knockouts indicates that a stimulus for UB outgrowth which “bypasses” canonical GDNF-Ret signaling is active in these mice (Fig. 2). To investigate this, global gene expression patterns were compared between wildtype and Ret^(−/−)^ kidneys isolated shortly after the beginning of kidney development (Fig. 2). Among the genes upregulated in the knockout kidney compared to the wild-type were a subset of FGFs, including FGF7 (a finding which was confirmed by qRT-PCR) (Table 1). The upregulation of FGFs in these rudimentary kidneys from Ret^(−/−)^ embryos not only raise the possibility that a FGF-dependent bypass pathway might play an integral role in GDNF-Ret-independent budding, they also support the notion that FGF-mediated GDNF-independent WD budding (Maeshima et al., 2007; Rosines et al., 2007; Choi et al., 2009; Tee et al., 2010) is a good in vitro model system in which to investigate the bypass pathway.

**Table 1.**
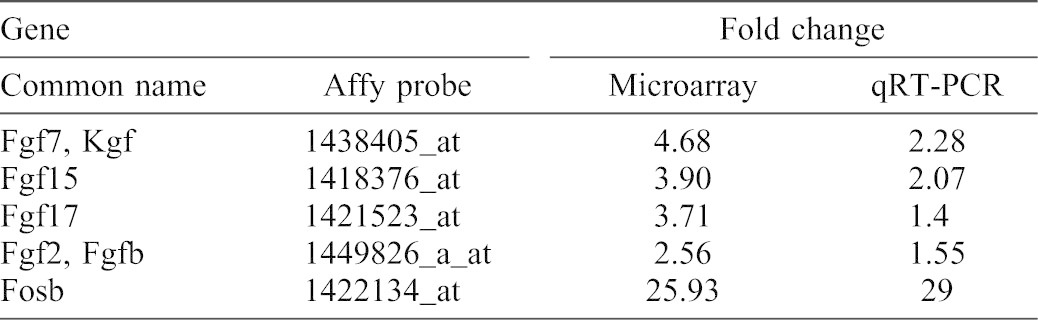
Expression of select genes in Ret^(−/−)^ versus Ret^(+/+)^ kidneys.

### GDNF-independent budding of the WD is mediated by AKT activation independent of PI3K

RTKs, such as Ret and the FGF receptors, represent an important class of receptors which (upon binding of their ligands) can activate a variety of intracellular signaling cascades, including the RAS/extracellular signal-regulated kinase (MEK/ERK), phosphatidylinosityol 3-kinase (PI3K)/Akt, p38 mitogen activated protein kinase (p38-MAPK), and c-Jun N-terminal kinase (JNK) pathways (Takahashi, 2001). Among these various signaling cascades, PI3K/Akt signaling appears key to GDNF-dependent outgrowth of the UB. For example, it has been shown that GDNF-mediated Ret activation increases PI3K activity and the phosphorylation of Akt in Ret-expressing MDCK cells (Tang et al., 2002). In addition, inhibition of PI3K activity, but not that of MEK/ERK or p38-MAPK, was found to block GDNF-dependent ectopic UB outgrowth in in vitro cultures of the entire region of intermediate mesoderm dissected from E10.5 mouse embryos (Tang et al., 2002).

**Fig. 3.**
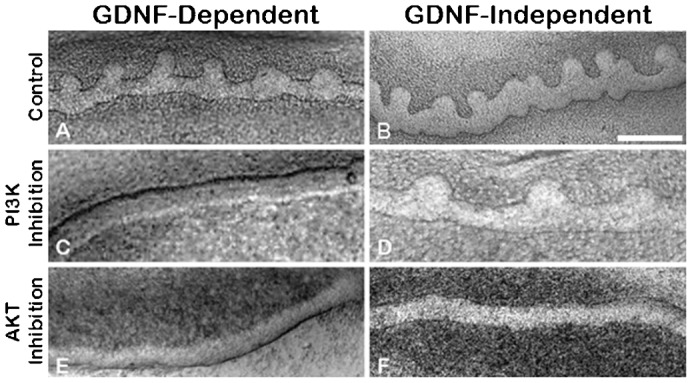
PI3-kinase independent activation of Akt is key to UB outgrowth in GDNF-independent budding. Phase-contrast photomicrographs of WDs induced to bud in the presence of 125 ng/ml of GDNF (GDNF-dependent) or absence of GDNF (GDNF-independent). (**A**,**B**) Control WDs, (**C**,**D**) PI3K signaling inhibition with the addition of 20 µM LY294002 inhibited GDNF-dependent budding (C) but did not affect GDNF-independent budding (D). (**E**,**F**) Akt inhibition with 5 µM Akt inhibitor IV inhibited both GDNF-dependent and GDNF-independent budding. No evidence of budding was seen with the addition of the inhibitors in 3 or more independent cultures. Scale bar: 200 µm.

However, downstream signaling events have only recently been examined in GDNF-independent budding. For example, in cultures of whole mesonephros, we found that, in addition to activation of PI3K/Akt signaling, GDNF-independent WD budding also leads to the activation of MEK/ERK signaling (Table 2) (Maeshima et al., 2007). In this study, we utilized the in vitro isolated WD culture system to probe intracellular signaling pathways potentially involved in GDNF-independent WD budding. As expected, inhibition of PI3K signaling (but not p38 MAPK or MEK/ERK signaling) in isolated WDs cultured in the presence of GDNF blocked UB emergence from the WD (Fig. 3; Table 3). However, the same effect was not seen in GDNF-independent budding conditions with the same PI3K inhibitor. In this case, perturbation of PI3K had no effect on budding (Fig. 3; Table 3), however inhibition of AKT activity blocked WD budding in GDNF-independent budding (Fig. 3; Table 3). In fact, perturbation of AKT activity blocked budding in both GDNF-dependent and GDNF-independent budding. As the PI3K pathway is generally considered to be common to the activation of AKT (Brugge et al., 2007; Mahajan and Mahajan, 2012), the data suggest that GDNF-independent budding involves signaling pathways which mediate activation of AKT without activation of PI3-kinase – i.e. GDNF-independent budding involves PI3K-independent AKT activation.

**Table 2.**
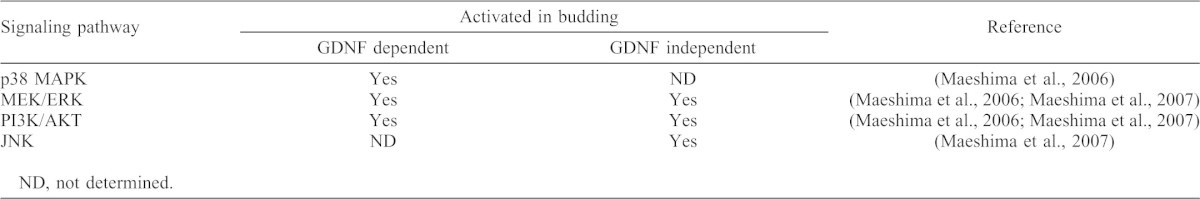
Signaling pathways activated in WD budding.

**Table 3.**
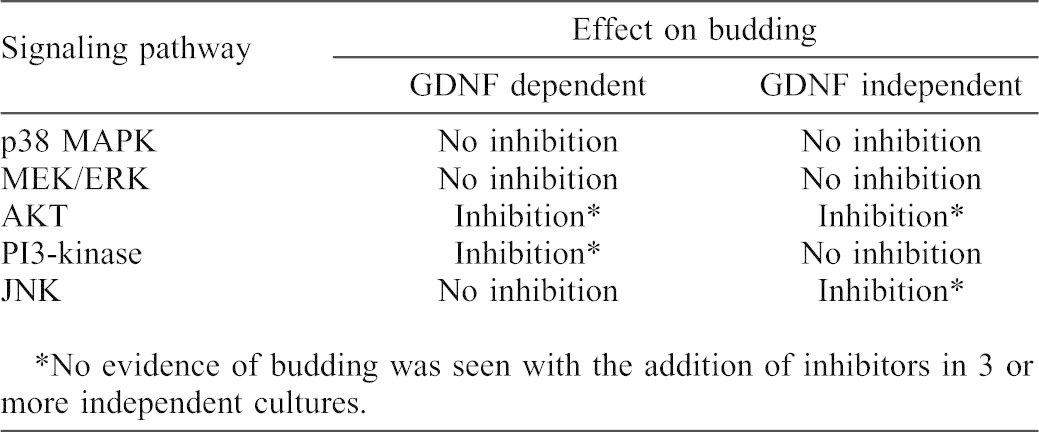
Inhibitors of signaling pathways.

### GDNF-independent budding is mediated by JNK signaling

**Fig. 4.**
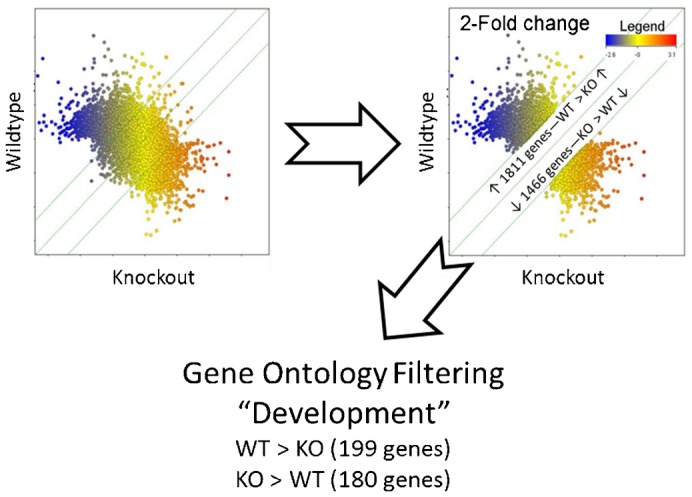
Genetic expression analysis of Ret^(−/−)^ compared to wild-type mice revealed differentially patterns of gene expression. Microarray comparison of gene expression between the wildtype and Ret knockout kidneys are displayed by scatter-plot and colored according to expression on the Ret knockout arrays. 1466 genes were upregulated 2-fold or greater in the knockout kidneys and 1811 were upregulated 2-fold or greater in the wildtype kidneys. These genes were further filtered based on the Gene Ontology annotation of “development” (GO:0007275) resulting in 199 genes increased in the wildtype and 180 genes increased in the knockout.

**Fig. 5.**
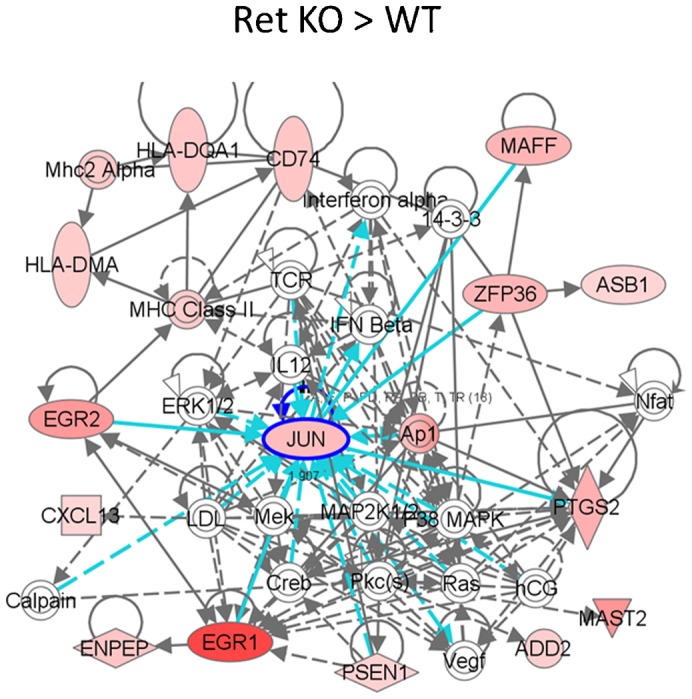
Pathway analysis of genes expressed higher in mutant mice revealed networks of interacting genes. The 180 genes expressed ≥2-fold higher in the Ret knockout kidney compared to the wildtype kidney were processed by IPA into several networks, one of which demonstrated a JUN hub. Solid lines represent direct interaction; dashed lines represent indirect interactions. Blue lines indicate those interactions involving JUN.

We have previously shown that in addition to AKT and ERK activation, GDNF-independent outgrowth of the UB also activates the JNK pathway (Maeshima et al., 2007) (Table 2), suggesting that this signaling pathway plays a role in WD budding in the absence of GDNF. Supporting this notion, pathway analysis of the 180 developmentally annotated genes with increased expression in the Ret^(−/−)^ kidney versus the wildtype (Fig. 4) resulted in several networks one of which demonstrated the existence of a signaling hub for the Jun oncogene (Fig. 5). Taken together with the fact that c-Jun N-terminal kinases (JNKs) have been reported to be capable of activating Akt signaling independent of PI3K (Shao et al., 2006; Chaanine and Hajjar, 2011), the role of the JNK signaling pathway in GDNF-independent budding was investigated.

### FosB regulates GDNF-independent WD budding

**Fig. 6.**
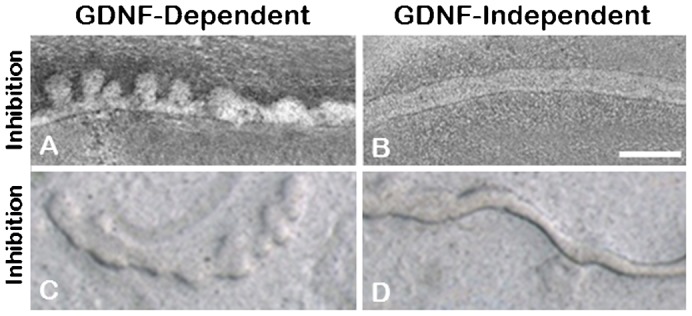
JNK activation and assembly into AP-1 transcription factor complex is key to UB outgrowth in GDNF-independent budding. Phase contrast photomicrographs of isolated WDs induced to bud in the presence of either 125 ng/ml of GDNF (GDNF-dependent) or 125 ng/ml FGF7 and 500 ng/ml follistatin (GDNF-independent). The addition of 5 µM JNK inhibitor II blocked GDNF-independent budding (**A**), but not GDNF-dependent budding (**B**). Inhibition of AP-1 transcription factor activity, with the addition of 20 µM SR 11032 to the media, had no observable effect on GDNF-dependent budding (**C**), but suppressed budding under GDNF-independent conditions (**D**). No evidence of budding was seen with the addition of either inhibitor in 3 or more independent cultures. Scale bar: 200 µm.

Inhibition of JNK-mediated signaling selectively blocked WD budding in the absence of GDNF, but not in its presence (Fig. 6). JUN family members can dimerize with other proteins to form the AP-1 transcription factor complex (Eferl and Wagner, 2003). Inhibition of AP-1 transcription factor activity (with SR11032) similarly inhibited GDNF-independent WD budding but not GDNF-dependent budding (Fig. 6). Thus along with PI3K-independent Akt activation, both JNK signaling and AP-1 activation appear to play key roles in GDNF-independent WD budding.

**Fig. 7.**
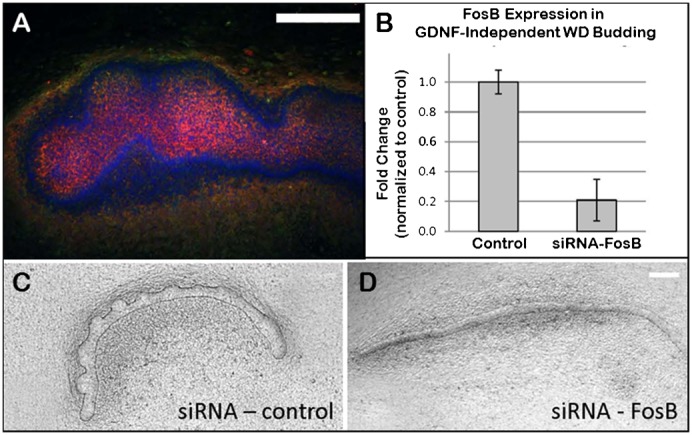
Localization and effect of inhibition of FosB expression in GDNF-independent WD budding. (**A**) Immunohistochemistry for FosB in the budded WD. Red  =  FosB; green  =  E-cadherin; blue  =  DAPI. (**B–D**) Suppression of FosB expression was accomplished by the transfection of small interfering RNA (siRNA) against FosB in the cultured WD. (B) Quantitative real-time PCR verified a near-80% reduction in FosB expression in the WD with siRNA transfection. (C,D) Phase contrast photomicrographs of isolated WDs cultured in the absence of GDNF, but in the presence of 125 ng/ml FGF7 and 500 ng/ml follistatin. Inhibition of FosB expression resulted in the inhibition of GDNF-independent WD budding. No evidence of budding was seen with the transfection of the siRNA in 3 or more independent cultures. Scale bars: 200 µm.

In addition to the JUN protein family, the AP-1 complex is also composed of members of the Fos, ATF (activating transcription factor) and MAF (musculoaponeurotic fibrosarcoma) protein families (Eferl and Wagner, 2003). Importantly, the gene displaying the highest expression in the knockout relative to the wild-type was FosB, a finding validated by qRT-PCR (Table 1). Immunohistochemical analysis using an anti-Fosb antibody confirmed the presence of FosB in isolated WDs displaying GDNF-independent budding (Fig. 7). Furthermore, suppression of FosB expression in the WD using small interfering RNA inhibited GDNF-independent budding, but not GDNF-dependent budding (Fig. 7). Collectively, these results strongly support a role for the JNK/FosB-AP-1 signaling pathway in mediating GDNF-independent budding of the WD.

**Fig. 8.**
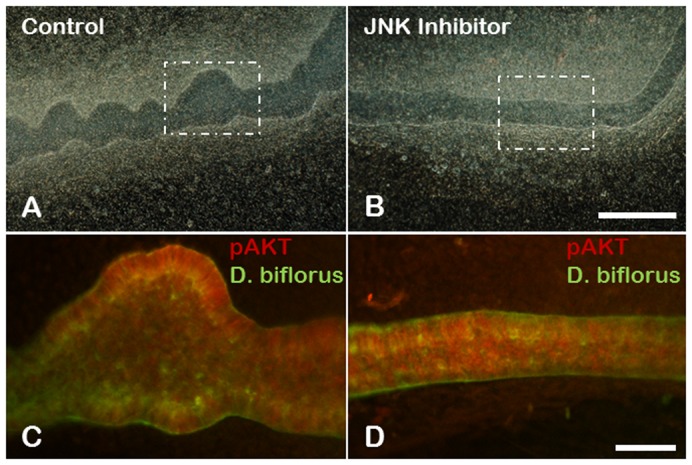
Inhibition of JNK does not block activation of Akt in GDNF-independent WD budding. (**A**,**B**) Darkfield photomicrogrpahs of isolated WDs induced to bud in the presence of 125 ng/ml FGF7 and 500 ng/ml follistatin with (A) or without (B) 20 µM JNK inhibitor. Scale bar: 200 µm. (**C**,**D**) Confocal fluorescent photomicrographs showing localization of phospho-Akt (pAkt) in WDs cultured under GDNF-independent budding conditions with or without JNK inhibitor. No evidence of budding was seen with the addition of the inhibitor in 3 or more independent cultures. Red-pAkt; green-Dolichos biflorus lectin. Scale bar: 25 µm.

Data from other organs have revealed a role for the JNK-signaling pathway in PI3K-independent activation of Akt (Shao et al., 2006; Chaanine and Hajjar, 2011), raising the possibility that JNK is activating Akt in GDNF-independent WD budding. To investigate this possibility further, the presence of phosphorylated Akt (pAkt) was examined in isolated WDs cultured under GDNF-independent WD budding conditions in the presence and absence of JNK inhibitor (Fig. 8). Immunohistochemical analysis using an anti-pAkt antibody revealed the presence of activated Akt even in the presence of 20 µM JNK inhibitor (Fig. 8). Thus, in the developing kidney activation of Akt in GDNF-independent budding was independent of JNK activity.

## Discussion

We sought to provide mechanistic insight into how animals without *Ret*, *Gdnf*, or *Gfra1* form a ureteric bud and rudimentary kidneys 20–50% of the time (Schuchardt et al., 1994; Moore et al., 1996). Employing a combination of global gene expression analysis of embryonic kidneys from Ret^(−/−)^ animals and ex vivo wet-lab analyses using a well-established ex vivo model of WD budding (Maeshima et al., 2007; Rosines et al., 2007; Choi et al., 2009; Tee et al., 2010), we found that: 1) perturbation of PI3K inhibited GDNF-dependent, but not GDNF-independent WD budding; 2) blockade of AKT signaling inhibited WD budding in both conditions; 3) a signaling hub for the Jun oncogene exists in GDNF-Ret independent budding and that perturbation of this pathway (by blocking either c-Jun N-terminal kinases (JNKs) or the AP-1 complex) selectively inhibited GDNF-independent budding; 4) the most highly differentially expressed gene in the Ret^(−/−)^ hypomorphic kidney was the c-Jun binding partner, FosB; 5) siRNA-mediated suppression of FosB selectively inhibited GDNF-independent WD budding; and 6) activation/phosphorylation of AKT in GDNF-independent WD budding is independent of c-Jun mediated signaling. Taken together, the data suggest that GDNF-Ret independent UB outgrowth is likely to be due to signaling cascades requiring activation of AKT independent of both PI3K and the JNK/FosB-AP-1 signaling complex.

Here, a well-established ex vivo model of WD budding was employed to analyze GDNF-independent budding in comparison to GDNF-dependent budding. A number of FGFs were upregulated in the kidneys of mutant animals compared to the wildtype (Table 1). Although a recent study demonstrated the expression of FGF8 and FGF10 in human WD epithelial and mesenchymal cells (Carev et al., 2008), there is little information on the expression of FGFs in kidney development during these very early stages of kidney development. Nevertheless expression analysis has been performed on later stages of kidney development subsequent to UB outgrowth which supports the observations presented here. For example, a recent examination of the GUDMAP database revealed the expression of several FGFs in the early wildtype kidney, including 1, 7, 8, 9, 10, 12, and 20 (Brown et al., 2011). In addition, FGF receptors (Fgfr) appear to be appropriately expressed at this developmental time point and recent data indicates that deletion of Fgfr2 (the receptor for FGF7 and FGF10) from the stromal cells surrounding the WD results in perturbed induction of the ureteric bud (Walker et al., 2013). Thus, data support the notion that the expression of various FGFs may serve as compensatory factors mediating signaling mechanism(s) necessary for the formation of the UB in the absence of canonical GDNF-Ret signaling (Chi et al., 2004; Michos et al., 2010; Pitera et al., 2012). For example, FGF7, which is upregulated in the ret knockout when budding manages to occur and a rudimentary kidney forms (Maeshima et al., 2007), as well as FGF2 and FGF10, is capable of inducing ectopic bud formation in WDs expressing human Sprouty2 (Spry2, a negative regulator of receptor tyrosine kinase signaling) (Chi et al., 2004). In addition, kidney agenesis can be rescued in either Ret^(−/−)^ or Gdnf^(−/−)^ mice by crossing these mutant strains with mice deficient in Spry1, which is believed to allow normal kidney organogenesis through a mechanism dependent on FGF10 (Michos et al., 2010). Thus, as with the in vitro/ex vivo data, in vivo data support the notion that the expression of FGFs may be serving as a compensatory mechanism for activating signaling pathways to form the UB in the absence of Gdnf-Ret signaling.

A reduction in BMP/Activin signaling activity also appears to be important, and this is supported by in vivo and ex vivo data (Maeshima et al., 2006; Maeshima et al., 2007; Choi et al., 2009; Tee et al., 2010). Such modulation of the BMP/Activin pathway has been shown to play a role in in vivo UB emergence in mice (Michos et al., 2007). For instance, Six1 knockout mice display renal agenesis despite apparently normal levels of GDNF mRNA (Kreidberg et al., 1993; Xu et al., 2003). In addition, recent evidence indicates that Six1 also regulates the expression of Grem1, an antagonist of Bmp4 (Nie et al., 2011), a factor which suppresses GDNF activity (Miyazaki et al., 2000; Brophy et al., 2001). Treatment of renal tissues isolated from Grem1 knockout animals with recombinant grem1 protein induced UB outgrowth (Michos et al., 2007). Thus, while GDNF appears to be the predominant soluble growth factor involved, it is becoming increasingly clear that this critical morphogenetic process is modulated by an interplay of stimulatory and inhibitory growth factors (Bush et al., 2004; Maeshima et al., 2006).

Inhibitors of various signaling pathways demonstrated that Akt activation was key to the emergence of the epithelial bud in both GDNF-dependent and GDNF-independent budding. However, in the case of GDNF-independent budding, activation of Akt was apparently via a PI3K-independent mechanism since inhibition of PI3K did not hinder budding in the absence of GDNF (Fig. 3). Examination of a number of other potential signaling pathways implicated the JNK/AP-1 signaling pathway as playing a potential role in GDNF-independent WD budding. Microarray expression analysis also found that FosB (which can dimerize with c-Jun to form the AP-1 transcription factor complex) was the most highly differentially expressed gene in the Ret^(−/−)^ metananephroi (Table 1), but its potential role in the developing kidney has remained largely unexplored. FosB has been implicated in the regulation of cell proliferation and differentiation in other organ systems (Haasper et al., 2008). Moreover, in the brain, increased FosB expression has been demonstrated in Gdnf^(+/−)^ mutant mice and has been associated with increased dendritic branching (Airavaara et al., 2004; Kim et al., 2009). Treatment of isolated WDs with either siRNA against FosB (Fig. 7) or an inhibitor of the AP-1 transcription factor complex (Fig. 6) supported the notion that GDNF-independent WD budding was dependent upon FosB/Jun/AP-1 signaling. Although the direct stimulant for the JNK pathway remains unclear, FGFs have been implicated in JNK signaling in other systems. For example, in alveoli, the effects of FGF7 on genes can be arrested by JNK inhibition (Chang et al., 2005; Qiao et al., 2008). Moreover, exogenous in vivo administration of FGF15 has been shown to activate JNK in the livers of mice genetically modified for the study of bile-acid synthesis (Kong et al., 2012).

**Fig. 9.**
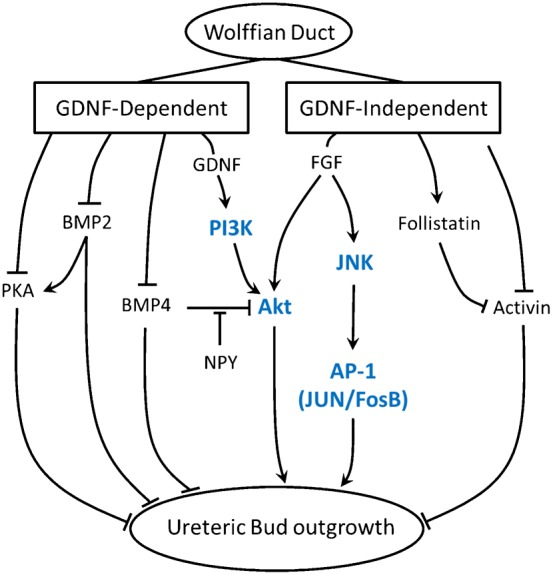
Proposed signaling process for GDNF-independent UB outgrowth. A possible schema for the signaling processes involved in GDNF/Ret-independent budding of the Wolffian duct, incorporating the study's *in vitro* pathway findings and existing knowledge. Arrowheads indicate stimulatory signal. T-capped lines indicate inhibitory signal. Observations from the results of this study are highlighted in blue. A role for BMP4, PKA and activin in budding regulation has been previously established (Miyazaki et al., 2000; Maeshima et al., 2007; Tee et al., 2010). PI3K  =  phosphoinositide 3-kinase; PKA  =  protein kinase A; BMP4  =  bone morphogenetic protein 4; JNK  =  Jun N-terminal kinases; FosB  =  FBJ murine osteosarcoma viral oncogene homolog B; AP-1  =  activator protein-1 transcription factor.

In summary, although both GDNF-dependent and GDNF-independent budding from the WD *ex vivo* require RTK and Akt activation, GDNF-dependent budding requires PI3K activation while GDNF-independent budding appears to require PI3K-independent activation of Akt, as well as JNK/Fosb signaling. The data indicate that both of these signaling pathways are necessary, but neither is sufficient on its own for GDNF-independent budding. The accumulated data on signaling pathways is summarized in Table 3. By adding these new results to previously obtained data on BMP4 (Miyazaki et al., 2000), protein kinase A (Tee et al., 2010), neuropeptide Y (NPY) (Choi et al., 2009) and activin (also studied in Ret knockout kidneys (Maeshima et al., 2007)), a revised network for GDNF-independent budding has been generated (Fig. 9).
